# Development and Validation of LC-MS/MS for Analyzing Potential Genotoxic Impurities in Pantoprazole Starting Materials

**DOI:** 10.1155/2020/6597363

**Published:** 2020-03-09

**Authors:** Yuyuan Chen, Song Wu, Qingyun Yang

**Affiliations:** Institute of Materia Medica, Chinese Academy of Medical Sciences & Peking Union Medical College, Beijing 100050, China

## Abstract

Pantoprazole sodium (PPZS) is a selective proton pump inhibitor used in the prevention and treatment of gastric acid-related diseases. Six potentially genotoxic impurities (PGIs) are involved in 5-difluoromethoxy-2-mercapto-1H-benzimidazole (DMBZ), which is the starting material of PPZS. To date, no suitable method has yet been developed for PGI separation and quantification at the threshold of toxicological concern levels. In this study, a sensitive and reliable liquid chromatography-tandem mass spectrometry method was developed and validated for the quantitative analysis of six PGIs in DMBZ according to the guidelines of the International Council for Harmonization (ICH). The calibration curves showed good linearity within the studied range, and the correlation coefficient of fitting exceeded 0.998 for each impurity. The sensitivity of the proposed method was in the range of 0.6–10.0 ng/mL. Good recoveries were observed in the range of 94.32%–107.43% with RSD values below 6.5%. Quantitative analysis of impurities in substance batches of DMBZ showed the high efficiency of the developed method at a low level. Hence, the proposed method is practical and useful in the detection and qualification of PGIs in DMBZ and may be applied to ensure the safe use of PPZS in clinical treatment.

## 1. Introduction

**Figure 1:**
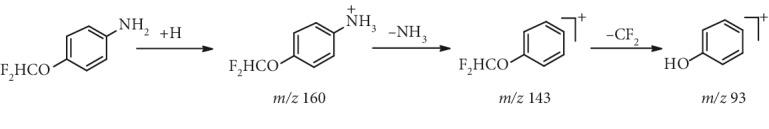
Chemical structures of PPZS, DMBZ, and potential genotoxic impurities.

Pantoprazole sodium (PPZS, Figure 1) is a selective and long-acting proton pump inhibitor clinically used for the short-term treatment of erosive esophagitis and ulceration associated with gastroesophageal reflux disease and other conditions involving excess stomach acid, such as Zollinger–Ellison syndrome [1–3]. As a racemic mixture, PPZS is available in intravenous, tablet, and granule formulations. PPZS (Pantoloc® i.v.) from Nycomed GmbH is highly tolerated by patients and can be administered through intravenous infusion. Pharmaceutical factories in several countries are approved for PPZS production, and preparations of the drug have achieved significant economic and social benefits.

**Figure 2:**
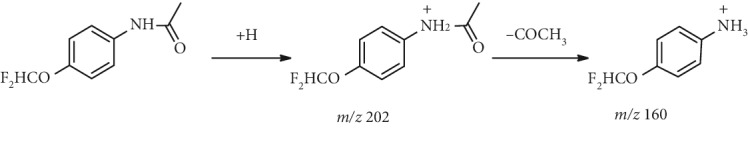
Schematic diagram of DMBZ and potential genotoxic impurities.

**Figure 3:**
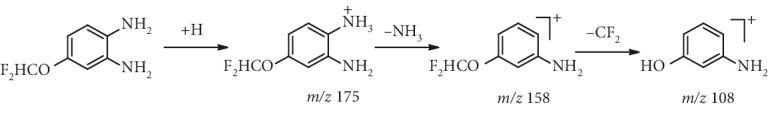
Typical MRM chromatograms of a mixed solution of impurities C–H and DMBZ (100 ng/mL).

Two potentially genotoxic impurities (PGIs), namely, 2-chloromethyl-3,4-dimethoxy-pyridine hydrochloride (impurity A) and pantoprazole sulfone N-oxide (impurity B), with structurally alerting functional groups [4], have been detected during PPZS synthesis. Six PGIs (impurities C–H) involved in the synthesis of the starting material of PPZS and 5-difluoromethoxy-2-mercapto-1H-benzimidazole (DMBZ) have also been detected. Among these impurities, impurities D–G are reaction intermediates, while impurities C and H are by-products (Figures 2 and 3). The detection and quantification of such impurities during drug production is remarkably challenging. PGIs can induce chromosomal breaks, genetic mutations, or rearrangements in mammalian cell systems [5–7]. Impurities remarkably affect the purity of the starting material and even the final drug substance. Completely eliminating PGIs from the pharmaceutical product is impossible. Thus, reduction of impurities to the lowest possible level in active pharmaceutical ingredients (APIs) and starting materials is crucial. Therefore, a new and valid method for the detection and quantification of trace impurities must be developed.

Several reviews on the control of genotoxic impurities have been published [8–16]. The presence of potential PGIs has also attracted the attention of regulatory authorities, and relevant guidelines have been released to the pharmaceutical industry [17–19]. These guidelines propose a threshold of toxicological concern (TTC) of 1.5 *μ*g/day for drug formulations, and the permitted limit for PGIs is set on the basis of the maximum daily dose of the drug. For example, for PPZS with a maximum daily dosage of 80 mg, the estimated permitted level of these impurities is 10 ppm. The quantitative determination of PPZS and its related impurities using high-performance liquid chromatography (HPLC) is established in the European Pharmacopoeia, the United States Pharmacopeia, and the Pharmacopoeia of the People's Republic of China [20–22]. However, the major disadvantage of HPLC for detecting these PGIs at the 10 ppm level is the low sensitivity of the UV detector, which cannot meet the needs of detection and quantification. Several sophisticated techniques, such as LC-mass spectrometry (MS) and gas chromatography (GC)-MS, have been used to quantify PGIs, including impurities A and B, in PPZS APIs [23–25]. Moreover, a number of GC-MS and UPLC-MS/MS methods for the separation and quantification of similar types of PGIs in bulk drugs and their corresponding formulations, such as rabeprazole, atenolol, darunavir, and ritonavir, have been published [26–29]. Nevertheless, to the best of our knowledge, a suitable method for the simultaneous separation and quantification of these six PGIs at the TTC level in the starting material of PPZS, i.e., DMBZ, has not been reported. The detection and quantification of impurities, especially genotoxic impurities, in starting materials and APIs is a mandatory requirement implemented by regulatory authorities [30]. The presence of PGIs in starting materials may exert a significant impact on the quality and safety of PPZS APIs. Thus, developing a sensitive and validated method is required for the reliable estimation of PGIs in DMBZ and commercialization of APIs.

In this research, a new and sensitive LC-MS/MS method with adequate limit of quantification (LOQ) values was established and validated for the quantitative determination of PGIs in DMBZ. The reported methods are validated according to International Council for Harmonization (ICH) guidelines in terms of LOQ, specificity, accuracy, precision, and linearity [31]. In the present study, the LC/MS/MS method was used for the quantification of six PGIs in DMBZ due to its high selectivity and sensitivity. Our results are predicted to be significant for the safe use of this API for the long-term clinical prevention and treatment of gastric acid-related diseases.

## 2. Materials and Methods

### 2.1. Materials

The DMBZ bulk (batch nos. 20130323, 20130519, 20141001, 20180903, 20181001, and 20181003) was produced in Chizhou Dongsheng Pharmaceutical Co., Ltd. The six PGIs (impurities C–H) were obtained from Shenyang Manwei Chemical Co., Ltd. The purities of the compounds (>99%) used in this study were examined by using the appropriate HPLC methods. Ammonium acetate and formic acid were supplied by TEDIA Company, Inc. (Fairfield, OH, USA), and all reagents were HPLC-grade with ≥99% purity. MS-grade methanol was purchased from Mallinckrodt Baker Inc. (Phillipsburg, NJ, USA) and used to prepare the mobile phases. Ultrapure water was prepared from a Milli-Q water purification system (Bedford, MA, USA). The distilled water used was Wahaha purified water.

### 2.2. Instrumentation

An Agilent 1290 series HPLC system and a 6410B triple quadrupole MS (Agilent Technologies, Inc.; Santa Clara, CA, USA) equipped with an electrospray ionization device were used for sample analysis. An Alltima C18 column (150 × 4.6 mm I.D., 5 *μ*m) was purchased from W.R. Grace & Co. (Columbia, MD, USA).

The Alltima C18 column was operated at an oven temperature of 40°C for separation. Mobile phase A was 0.005 mol/L ammonium acetate aqueous solution containing 0.1% formic acid, and mobile phase B was methanol. Mobile phases A and B were mixed at a ratio of 60 : 40 (V/V). The flow rate and injection volume were 0.5 mL/min and 10 *μ*L, respectively.

A triple quadrupole MS equipped with a positive electrospray ionization source was used in the MRM mode. The equipment was set with a drying gas flow, nebulizer pressure, gas temperature, and spray voltage of 10 L/min, 30 psi, 300°C, and 5500 V, respectively. A spray voltage of 5500 V was used for MS.

The MRM conditions were individually optimized for each of the six PGIs (impurities C–H) on account of their different structures, and the MS conditions for MRM are summarized in Table 1.

### 2.3. Preparation of Standard and Sample Solutions

The sample DMBZ solution was prepared at a high concentration of 20 mg/mL in methanol to evaluate the content of impurities C–H and assess p-GTI levels. Stock standards of the analytes (impurities C–H) were prepared at a concentration of 2 mg/mL in methanol. Subsequently, standard mixture solutions containing the six impurities at a concentration of 200 ng/mL (equivalent of 10 ppm) in methanol were obtained by diluting the stock standards for analysis in DMBZ batches.

### 2.4. Method Validation

#### 2.4.1. Linearity

To establish linearity, we prepared a calibration plot by analyzing six solutions in the concentration range of 20–1000 ng/mL. Approximately 20 mg of impurities C–H was accurately weighed, transferred to a 10 mL volumetric flask, and dissolved and diluted with methanol to volume. This solution was used as the stock standard solution and diluted step by step. Among the impurities, G was gradually diluted to concentrations of 1000, 500, 200, 100, and 50 ng/mL due to its slightly lower sensitivity compared with the other impurities. Impurity E was diluted stepwise to concentrations of 500, 200, 100, 50, and 20 ng/mL due to saturation at 1000 ng/mL. The remaining impurities were diluted to concentrations of 1000, 500, 200, 100, 50, and 20 ng/mL. The intercept, slope, and correlation coefficient were determined by linear regression data analysis and fitting to a linear regression model with a weighting scheme of 1/*x*.

#### 2.4.2. Limit of Quantification and Limit of Detection

Precisely measure the appropriate amount of 20 ng/mL solution under the linearity and dilute with methanol quantitatively and stepwise if necessary. The diluted solutions were separately injected into the chromatograph. LOQs and LODs were defined as the concentrations that could be detected and yield signal-to-noise (S/N) ratios of 10 : 1 and 3 : 1, respectively.

#### 2.4.3. Accuracy

The accuracy of the LC-MS/MS method was evaluated through spiked recovery experiments by using three concentration levels. Sample DMBZ solutions (*n* = 3 per level) at the test concentration (20 mg/mL) containing six impurities at 160 (80% level), 200 (100% level), and 240 ng/mL (120% level) were prepared and analyzed by determining the back calculated in comparison with the nominal concentration of each impurity. Accuracy was reported as percentage of mean recovery, and relative standard deviations (RSD%) were calculated for each concentration level.

#### 2.4.4. Precision and Stability

The precision of the proposed LC-MS/MS method was assessed by using standard mixture solutions (*n* = 6) containing six impurities at a concentration of 200 ng/mL. Solution stability was established by analyzing the standard mixture solutions at different time intervals (2, 4, 8, 12, and 24 h) at room temperature. Each sample was measured thrice, and the results were estimated by calculating the RSD%.

## 3. Results and Discussion

### 3.1. Analytical Method Development

This work aimed to develop a sensitive and reliable LC-MS/MS method to determine PGIs in DMBZ. Separating DMBZ and its six PGIs is critical because of their similar structure and polarities (Figure 1). The baseline separation of impurities was prioritized. Hence, C8 and C18 stationary phases were used with different carbon loadings as part of the preliminary work. Various mobile phases, such as different proportions of acetonitrile–ammonium acetate and methanol–ammonium acetate solutions, were tested. Good peak separation was observed on the Alltima C18 column (150 × 4.6 mm I.D.; 5 *μ*m particle size). Impurities C–H were quantified in DMBZ using methanol–water with 0.005 mol/L ammonium acetate as the mobile phase.

Specific quantitative ions determined according to the chemical structure and MS splitting decomposition law of each impurity were used for qualitative and quantitative analyses. The MS fragmentation pathways and quantitative ions of each potential impurity are shown in Table 2. The MS parameters were optimized by using analytical software.

Given that sample solutions of DMBZ were used in the prohibitive concentration of the LC-MS/MS analysis, the two approaches require efficient chromatographic separation for each impurity from DMBZ. At the time range of the DMBZ elution, the mobile phase and their eluents were transformed into waste to protect the ESI source and provide favorable conditions for analysis.

### 3.2. Method Validation

The proposed method was validated according to the criteria of ICH guidelines [27], including specificity, linearity, LOD, LOQ, accuracy, precision, and solution stability.

#### 3.2.1. Specificity

The specificity of the method was evaluated by injecting blank and individual PGIs and DMBZ at a concentration of 200 ng/mL. The corresponding MRM chromatograms of impurities C–H and DMBZ are shown in Figure 3. The chromatograms show that the developed methods could successfully separate the PGIs from one another and from the main drug.

#### 3.2.2. Sensitivity

The LOD and LOQ of all PGIs were analyzed on the basis of *S*/*N* ratios of 3 : 1 and 10 : 1, respectively, by injecting diluted solutions with known concentrations. LODs and LOQs related to impurities C–H at 20.0 mg/mL are shown in Table 3. Among these impurities, G revealed the weakest response and, thus, had the lowest sensitivity; by contrast, impurity D indicated the highest sensitivity. These low LOQ values were considered satisfactory and adequate for the specific analysis.

#### 3.2.3. Linearity

The linearity of the method was evaluated at six different concentration levels for each impurity due to their different detection sensitivities, as shown in Table 3. The linear range was 22.6–564.5 ng/mL for impurity E because of its overload at 1000 ng/mL. This linearity was satisfactorily illustrated by using a six-point calibration graph. The slope, intercept, and regression coefficient were calculated by using least-squares linear regression analysis. A weighting scheme of 1/*x* was used on the basis of the best estimation of the back-calculated concentration of the calibrators.

#### 3.2.4. Accuracy

The accuracy of the method was evaluated through spiked recovery experiments. Authentic impurities C–H were spiked into 20.0 mg/mL DMBZ in triplicate using concentration levels of 80% (160 ng/mL), 100% (200 ng/mL), and 120% (240 ng/mL). Good recoveries in the range of 94.32%–107.43% with RSD values below 6.5% were achieved, as shown in Table 3.

#### 3.2.5. Precision and Solution Stability

Precision was examined by injecting six individual preparations of the standard mixture solution containing impurities at the limit level (200 ng/mL). The RSD% of area for each impurity was calculated. The solution stability of the impurities in the sample solution was established by analyzing the standard mixture solutions at different time intervals (2, 4, 8, 12, and 24 h) at room temperature. The method validation results summarized in Table 3 indicate that our established method can reliably quantify these PGIs in DMBZ.

### 3.3. Sample Analysis

The validated LC-MS/MS method was applied to measure the aforementioned PGIs in six batches of DMBZ samples. The test concentration of DMBZ was 20.0 mg/mL, and that of the standard mixture containing impurities C–H was 200 ng/mL. The results are listed in Table 4. The levels of all PGI impurities were below the defined acceptable TTC limits, thereby indicating that all impurities are well controlled.

## 4. Conclusion

A sensitive and reliable LC-MS/MS method was developed and validated according to ICH guidelines for the quantitative analysis of six PGIs in the starting material of PPZS, DMBZ. The new method was specific, precise, accurate, and linear within the assessed concentration range. The detection levels of the six PGIs were below 10 ng/mL, especially for impurity D at 0.6 ng/mL. Efficient chromatographic separation of each impurity from DMBZ was carried out. Using a switch valve to divert the mobile phase and eluents from the MS detector, the ESI source was protected and favorable conditions were provided for analysis. Quantitative analysis of the impurities in substance batches of DMBZ showed the high efficiency of this method at a low level. As a versatile and convenient technique, the proposed method is expected to be used in evaluations of the stability of DMBZ production and analysis of PGIs as a model. This method is also applicable to the in-process monitoring of impurities during pharmaceutical manufacturing. The LC-MS/MS method is indispensable to producers of PPZS as it can ensure low amounts of PGIs in the final API. Therefore, the results of this study will help ensure the safe use of APIs during clinical treatment.

## Figures and Tables

**Table 1:** Mass spectrometric conditions for the LC-MS/MS analysis of impurities C–H.

Compound	Precursor ion *m/z*	Product ion *m/z*	Fragmentor (V)	CE (eV)	EMV (+)	MS1 RES	MS2 RES
DMBZ	217.1	166.9	135	30	500	Wide	Unit
Impurity C	160.2	93.0	115	20	500	Wide	Unit
Impurity D	202.2	160.0	115	15	500	Wide	Unit
Impurity E	175.1	108.0	115	20	500	Wide	Unit
Impurity F	247.1	187.0	125	15	500	Wide	Unit
Impurity G	205.1	137.0	115	15	500	Wide	Unit
Impurity H	110.1	65.0	135	20	500	Wide	Unit

**Table 2:** MS fragment pathways and quantitative ions of PGIs.

No.	Code	MRM (*m*/*z*)	MS fragment pathways
1	Impurity C	*m*/*z* 160.2 ⟶ *m*/*z* 93.0	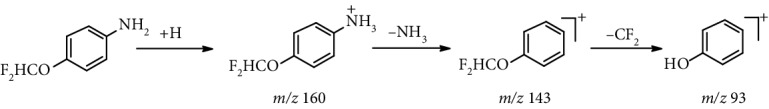
2	Impurity D	*m*/*z* 202.2 ⟶ *m*/*z* 160.0	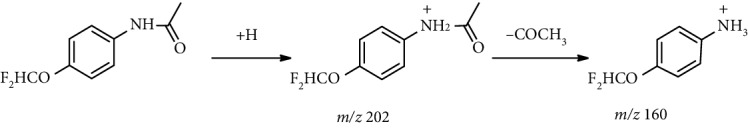
3	Impurity E	*m*/*z* 175.1 ⟶ *m*/*z* 108.0	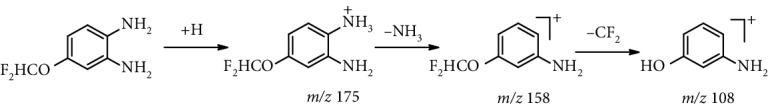
4	Impurity F	*m*/*z* 247.1 ⟶ *m*/*z* 187.0	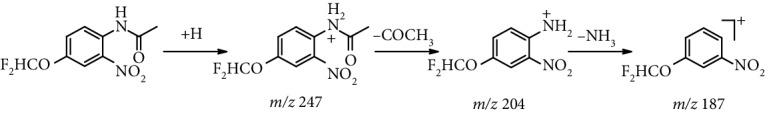
5	Impurity G	*m*/*z* 205.1 ⟶ *m*/*z* 137.0	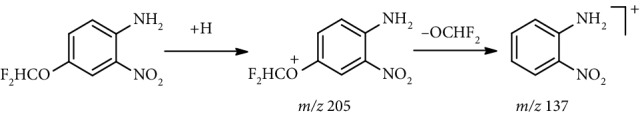
6	Impurity H	*m*/*z* 110.0 ⟶ *m*/*z* 65.0	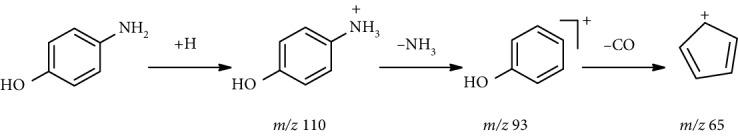

**Table 3:** Summary of the validation report of the LC-MS/MS method.

Parameter	Impurity C	Impurity D	Impurity E	Impurity F	Impurity G	Impurity H
Linear equation	*y* = 52.979*x* + 65.67	*y* = 11447*x* − 53046	*y* = 320.19*x* + 586.71	*y* = 541.39*x* − 3663.8	*y* = 15.745*x* + 60.04	*y* = 1174.6*x* + 1880.2
*R*	0.9998	0.9999	0.9988	0.9999	0.9991	1.0000
Linearity range (ng/mL)	20.8∼1042.0	20.3∼1013.0	22.6∼564.5	20.3∼1015.0	50.6∼1012.0	19.8∼988.0
Detection limit (ng/mL)	0.20	0.03	0.10	0.20	1.00	0.05
Quantitation limit (ng/mL)	1.00	0.10	0.50	1.00	5.00	0.20
Precision% (*n* = 6)	5.82	4.94	3.13	6.17	2.69	5.66
Stability (24 h)	4.91	7.37	1.89	4.47	6.67	4.16
Accuracy at 80% level (*n* = 3)						
% recovery	97.85	94.63	98.36	97.19	97.07	107.43
RSD% (*n* = 6)	3.67	6.35	3.50	5.83	4.86	1.96
Accuracy at 100% level (*n* = 3)						
% recovery	98.58	98.17	94.32	100.94	95.03	103.63
RSD% (*n* = 6)	5.06	5.45	3.19	4.85	4.62	0.94
Accuracy at 120% level (*n* = 3)						
% recovery	98.02	97.34	93.42	98.05	102.20	106.29
RSD% (*n* = 6)	4.05	4.11	3.07	3.35	4.36	1.78

**Table 4:** Amount of impurities C–H in starting material DMBZ.

PGIs (ng/mL)	Batch no					
	20130323	20130519	20141001	20180903	20181001	20181003
Impurity C	1.54	0.50^*∗*^	0.50^*∗*^	0.02^*∗*^	0.04^*∗*^	0.05^*∗*^
Impurity D	0.49	0.19	0.19	0.02^*∗*^	0.07^*∗*^	0.05^*∗*^
Impurity E	0.04^*∗*^	0.03^*∗*^	0.27^*∗*^	0.67	0.36^*∗*^	0.61
Impurity F	0.17^*∗*^	0.24^*∗*^	0.02^*∗*^	0.01^*∗*^	0.01^*∗*^	0.01^*∗*^
Impurity G	0.60^*∗*^	0.09^*∗*^	0.09^*∗*^	0.02^*∗*^	0.04^*∗*^	0.01^*∗*^
Impurity H	0.12^*∗*^	0.07^*∗*^	0.07^*∗*^	0.01^*∗*^	0.01^*∗*^	0.01^*∗*^

^*∗*^The calculated value is lower than the LOQ, for reference only.

## Data Availability

The data used to support the findings of this study are available from the corresponding author upon request.

## Supplementary Materials

Supplementary Materials The supporting information file includes information as described in the main articles, including the spectrum data of samples, the MRM chromatograms of method validation, and the MRM chromatograms for all samples.
