# Supplementary material for: Development and Validation of LC-MS/MS for Analyzing Potential Genotoxic Impurities in Pantoprazole Starting Materials
Source: J Anal Methods Chem. 2020 Mar 9;2020:6597363. doi: 10.1155/2020/6597363 (PMC7085375; doi:10.1155/2020/6597363)
Supplement: Supplementary Materials — The supporting information file includes information as described in the main articles, including the spectrum data of samples, the MRM chromatograms of method validation, and the MRM chromatograms for all samples. [file 6597363.f1.doc]

# Supporting Data

Development and Validation of LC-MS/MS for Analyzing Potential Genotoxic Impurities in Pantoprazole Starting Materials

Yuyuan Chen, Song Wu, Jinlan Zhang, Zhe Wang, and Qingyun Yang*

*Institute of Materia Medica, Chinese Academy of Medical Sciences & Peking Union Medical College, Beijing 100050, China.*

Correspondence should be addressed to Qingyun Yang; yqy@imm.ac.cn

Telephone: +86-010-8316-3542, Fax: +86-010-6301-7757

**Contents**

1. Sample characterization……....……………………………………………………...…………S1

2. NMR and ESI MS spectra of PPZS…………..…………………………….………………..…S2

3. NMR and ESI MS spectra of DMEBZ……..…………………….……………………….....…S3

4. MRM chromatograms of mixed solution of impurities C~H………….....…..……...…...….....S4

5. Linearity of impurities C ~ H………….....…..…………………………………..….……........S5

6. Precision of impurities C ~ H………….....…..…………………………………..….…...….....S7

7. Recovery of impurities C ~ H………….....…..…………………………………..….…...….....S8

8. Solution stability of impurities C ~ H at different time intervals………... …... …...….…......S10

9. Sensitivity of impurities C ~ H………….....…..………………………………..….…...….....S11

10. MRM chromatograms of DMEBZ sample (Batch No: 20130323) ……………..…….….....S12

11. MRM chromatograms of DMEBZ sample (Batch No: 20130519) ……………..…….….....S13

12. MRM chromatograms of DMEBZ sample (Batch No: 20141001) ………………..….….....S14

13. MRM chromatograms of DMEBZ sample (Batch No: 20180903) ………………..….….....S15

14. MRM chromatograms of DMEBZ sample (Batch No: 20181001) ………………..….….....S16

15. MRM chromatograms of DMEBZ sample (Batch No: 20181003) ………………..….….....S17

**1. Sample characterization**

**1.1** **Pantoprazole sodium (PPZS) 5-(difluoromethoxy)-2-[[(3,4-dimethoxy-2-pyridinyl)methyl]sulfinyl]-1*H*-Benzimidazole sodium**

^1^H NMR (500 MHz, DMSO-d_6_) *δ* 8.21 (d, *J* = 5.5 Hz, 1H), 7.42-7.45 (d, *J* = 8.5 Hz, 1H), 7.24 (d, *J* = 2.0 Hz, 1H), 7.07 (d, *J* = 6.0 Hz, 1H), 7.02 (t, *J* = 76.0 Hz, 1H) , 6.71-6.73 (dd, *J* = 2.5, 9.0 Hz, 1H), 4.65 (d, *J* = 13.0 Hz, 1H), 4.34 (d, *J* = 13.0 Hz, 1H) , 3.89 (s, 3H), 3.77(s, 3H). ^13^C NMR (125 MHz, DMSO-d_6_) *δ* 164.28, 158.29, 147.07, 146.62, 145.78, 144.58, 144.49, 144.18, 117.50, 117.50, 110.94, 107.84, 107.41, 60.90, 56.68, 55.88. (+)-ESI *m/z*: 406.1 [M+H]^+^.

**1.2 5-difluoromethoxy-2-mercapto-1H-benzimidazole (DMEBZ)**

^1^H NMR (500 MHz, DMSO-d_6_) *δ* 12.63-12.65 (d, 2H), 6.96 (t, 2H), 7.16 (d, 1H), 7.14 (t, 1H). (+)-ESI *m/z*: 185.3 [M+H]^+^.

**2. NMR and ESI MS spectra of PPZS**

**
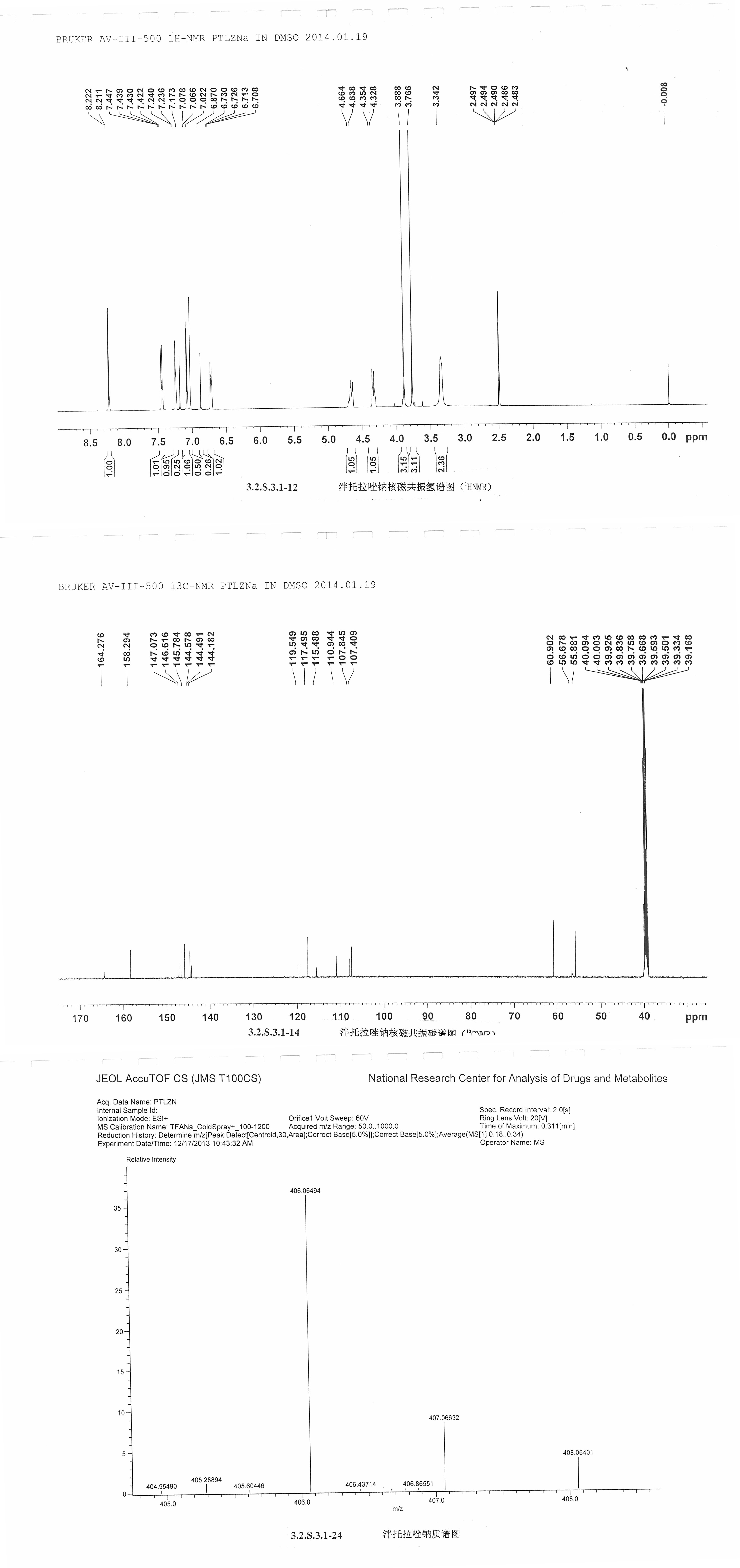
**

**3. NMR and ESI MS spectra of DMEBZ**

**
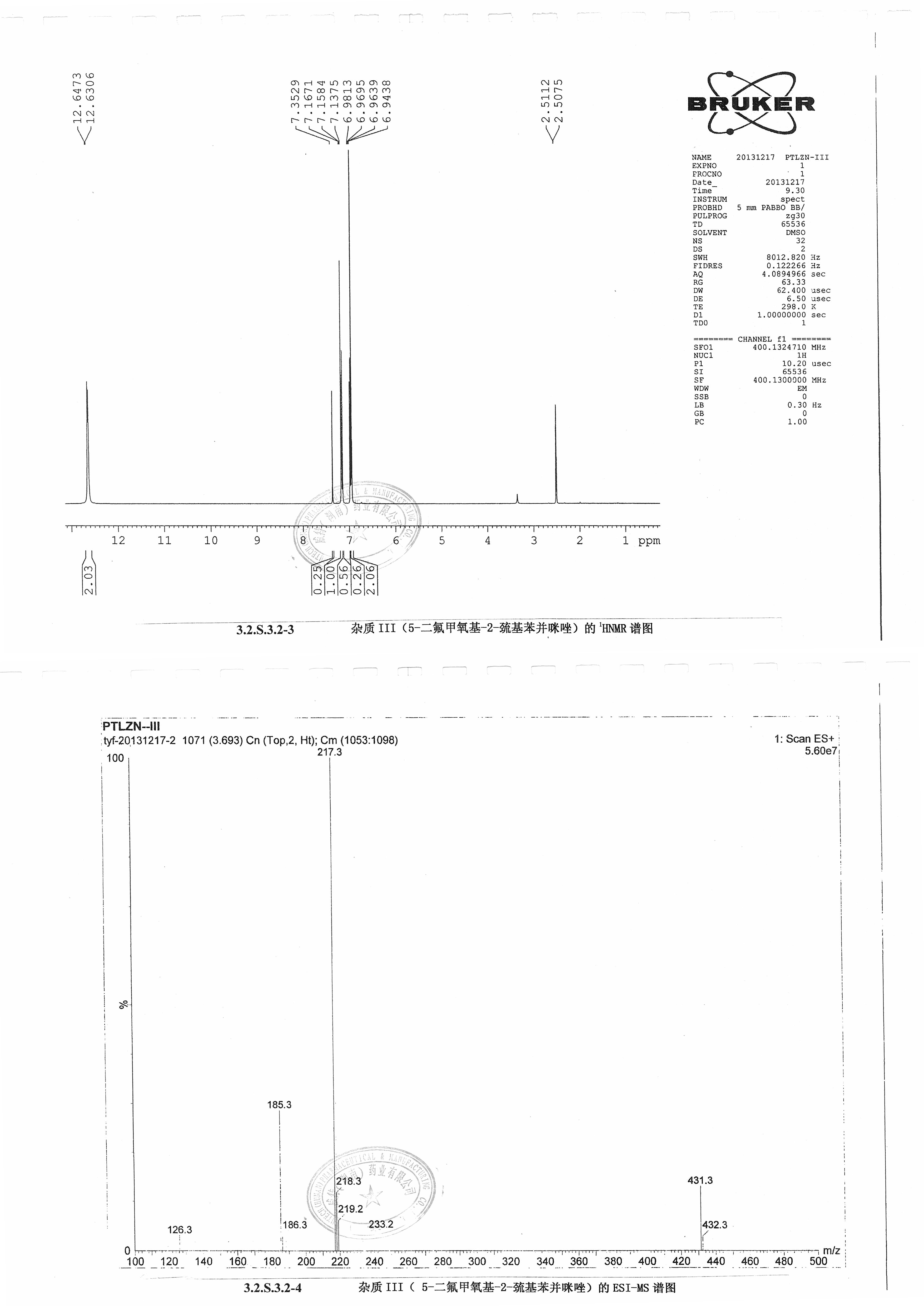
**

**4. MRM chromatograms of mixed solution of impurities C~H**

**5. Linearity of impurities C ~ H**.

|  |  |
| --- | --- |
|  |  |
|  |  |

**Linearity of impurity C**

| concentration（ng/ml） | 1042.0 | 521.0 | 208.4 | 104.2 | 52.1 | 20.8 |
| --- | --- | --- | --- | --- | --- | --- |
| Peak area-1 | 57964 | 28754 | 12631 | 6607 | 3643 | 1664 |
| Peak area-2 | 54575 | 26881 | 12326 | 6415 | 3798 | 1591 |
| Mean value | 56270 | 27817 | 12479 | 6511 | 3720 | 1628 |

**Linearity of impurity D**

| concentration（ng/ml） | 1013.0 | 506.5 | 202.6 | 101.3 | 50.7 | 20.3 |
| --- | --- | --- | --- | --- | --- | --- |
| Peak area-1 | 11946320 | 5854628 | 2266544 | 1187904 | 559761 | 245469 |
| Peak area-2 | 11261171 | 5424943 | 2148595 | 1037354 | 580917 | 219898 |
| Mean value | 11603745 | 5639785 | 2207570 | 1112629 | 570339 | 232683 |

**Linearity of impurity E**

| concentration（ng/ml） | 564.5 | 225.8 | 112.9 | 56.5 | 22.6 |
| --- | --- | --- | --- | --- | --- |
| Peak area-1 | 187063 | 72698 | 34340 | 21820 | 11066 |
| Peak area-2 | 178608 | 67889 | 30131 | 22589 | 8666 |
| Mean value | 182835 | 70294 | 32235 | 22205 | 9866 |

**Linearity of impurity F**

| concentration（ng/ml） | 1015.0 | 507.5 | 203.0 | 101.5 | 50.8 | 20.3 |
| --- | --- | --- | --- | --- | --- | --- |
| Peak area-1 | 564534 | 273057 | 104586 | 52280 | 24816 | 10566 |
| Peak area-2 | 529592 | 267789 | 98216 | 48947 | 26831 | 10010 |
| Mean value | 547063 | 270423 | 101401 | 50614 | 25823 | 10288 |

**Linearity of impurity G**

| concentration（ng/ml） | 1012.0 | 506.0 | 202.4 | 101.2 | 50.6 |
| --- | --- | --- | --- | --- | --- |
| Peak area-1 | 17850 | 8163 | 3327 | 2249 | 1215 |
| Peak area-2 | 15100 | 7707 | 3649 | 2194 | 1103 |
| Mean value | 16475 | 7935 | 3488 | 2222 | 1159 |

**Linearity of impurity H**

| concentration（ng/ml） | 988.0 | 494.0 | 197.6 | 98.8 | 49.4 | 19.8 |
| --- | --- | --- | --- | --- | --- | --- |
| Peak area-1 | 1195407 | 591638 | 239207 | 120864 | 60391 | 27994 |
| Peak area-2 | 1130287 | 570225 | 233472 | 106276 | 62494 | 24588 |
| Mean value | 1162847 | 580932 | 236340 | 113570 | 61442 | 26291 |

**6. Precision of impurities C ~ H**

|  |  |
| --- | --- |
|  |  |
|  |  |

| **Impurities** | **C** | **D** | **E** | **F** | **G** | **H** |
| --- | --- | --- | --- | --- | --- | --- |
| No. | Peak area | Peak area | Peak area | Peak area | Peak area | Peak area |
| 1 | 12705 | 2338525 | 60386 | 112411 | 3319 | 242679 |
| 2 | 11658 | 2194179 | 56114 | 109746 | 3255 | 231625 |
| 3 | 11594 | 2342055 | 60164 | 113086 | 3470 | 245051 |
| 4 | 12954 | 2450733 | 56925 | 122893 | 3411 | 243716 |
| 5 | 12550 | 2497519 | 59346 | 126961 | 3464 | 266308 |
| 6 | 13426 | 2494410 | 57261 | 124195 | 3471 | 266692 |
| Mean value | 12481 | 2386237 | 58366 | 118216 | 3399 | 249346 |
| RSD（%） | 5.82 | 4.94 | 3.13 | 6.17 | 2.69 | 5.66 |

**7. Recovery of impurities C ~ H**

| [reference substance](javascript:;) | [80%](javascript:;) level |
| --- | --- |
| [100%](javascript:;) level | [120%](javascript:;) level |

| **Impurity C** | **Samples** | **Added(ng)** | **Found (ng)** | **Recovery (%)** | **Mean value (%)** | **RSD (%)** |
| --- | --- | --- | --- | --- | --- | --- |
|  | 80% level | 1667.20 | 1742.89 | 104.54 | 97.85 | 3.67 |
|  |  | 1667.20 | 1611.83 | 96.68 |  |  |
|  |  | 1667.20 | 1594.17 | 95.62 |  |  |
|  |  | 1667.20 | 1653.94 | 99.20 |  |  |
|  |  | 1667.20 | 1582.57 | 94.92 |  |  |
|  |  | 1667.20 | 1602.45 | 96.12 |  |  |
|  | 100% level | 2084.00 | 2106.64 | 101.09 | 98.58 | 5.06 |
|  |  | 2084.00 | 1939.36 | 93.06 |  |  |
|  |  | 2084.00 | 2019.94 | 96.93 |  |  |
|  |  | 2084.00 | 2225.67 | 106.80 |  |  |
|  |  | 2084.00 | 2066.68 | 99.17 |  |  |
|  |  | 2084.00 | 1968.23 | 94.44 |  |  |
|  | 120% level | 2500.80 | 2541.73 | 101.64 | 98.02 | 4.05 |
|  |  | 2500.80 | 2319.05 | 92.73 |  |  |
|  |  | 2500.80 | 2482.99 | 99.29 |  |  |
|  |  | 2500.80 | 2575.91 | 103.00 |  |  |
|  |  | 2500.80 | 2412.70 | 96.48 |  |  |
|  |  | 2500.80 | 2375.91 | 95.01 |  |  |

| **Impurity D** | **Samples** | **Added(ng)** | **Found (ng)** | **Recovery (%)** | **Mean value (%)** | **RSD (%)** |
| --- | --- | --- | --- | --- | --- | --- |
|  | 80% level | 1620.80 | 1642.48 | 101.34 | 94.63 | 6.35 |
|  |  | 1620.80 | 1555.93 | 96.00 |  |  |
|  |  | 1620.80 | 1651.61 | 101.90 |  |  |
|  |  | 1620.80 | 1432.61 | 88.39 |  |  |
|  |  | 1620.80 | 1467.37 | 90.53 |  |  |
|  |  | 1620.80 | 1452.82 | 89.64 |  |  |
|  | 100% level | 2026.00 | 1892.73 | 93.42 | 98.17 | 5.45 |
|  |  | 2026.00 | 1920.40 | 94.79 |  |  |
|  |  | 2026.00 | 1997.70 | 98.60 |  |  |
|  |  | 2026.00 | 2193.37 | 108.26 |  |  |
|  |  | 2026.00 | 1935.85 | 95.55 |  |  |
|  |  | 2026.00 | 1993.57 | 98.40 |  |  |
|  | 120% level | 2431.20 | 2436.41 | 100.21 | 97.34 | 4.11 |
|  |  | 2431.20 | 2432.27 | 100.04 |  |  |
|  |  | 2431.20 | 2407.78 | 99.04 |  |  |
|  |  | 2431.20 | 2190.06 | 90.08 |  |  |
|  |  | 2431.20 | 2315.26 | 95.23 |  |  |
|  |  | 2431.20 | 2417.38 | 99.43 |  |  |

| **Impurity E** | | **Samples** | | **Added(ng)** | | **Found (ng)** | | **Recovery (%)** | **Mean value (%)** | **RSD (%)** | |
| --- | --- | --- | --- | --- | --- | --- | --- | --- | --- | --- | --- |
|  |  | 80% level | | 1806.40 | | 1752.88 | | 103.53 | 98.36 | 3.50 | |
|  |  |  |  | 1806.40 | | 1681.40 | | 99.31 |  |  |  |
|  |  |  |  | 1806.40 | | 1696.73 | | 100.22 |  |  |  |
|  |  |  |  | 1806.40 | | 1589.66 | | 93.90 |  |  |  |
|  |  |  |  | 1806.40 | | 1652.59 | | 97.61 |  |  |  |
|  |  |  |  | 1806.40 | | 1618.49 | | 95.60 |  |  |  |
|  |  | 100% level | | 2258.00 | | 2042.66 | | 96.50 | 94.32 | 3.19 | |
|  |  |  |  | 2258.00 | | 1928.55 | | 91.12 |  |  |  |
|  |  |  |  | 2258.00 | | 2064.48 | | 97.53 |  |  |  |
|  |  |  |  | 2258.00 | | 2011.16 | | 95.02 |  |  |  |
|  |  |  |  | 2258.00 | | 1907.53 | | 90.12 |  |  |  |
|  |  |  |  | 2258.00 | | 2024.45 | | 95.64 |  |  |  |
|  |  | 120% level | | 2709.60 | | 2322.54 | | 94.80 | 93.42 | 3.07 | |
|  |  |  |  | 2709.60 | | 2370.30 | | 95.28 |  |  |  |
|  |  |  |  | 2709.60 | | 2341.49 | | 97.18 |  |  |  |
|  |  |  |  | 2709.60 | | 2322.64 | | 91.93 |  |  |  |
|  |  |  |  | 2709.60 | | 2223.94 | | 89.21 |  |  |  |
|  |  |  |  | 2709.60 | | 2290.48 | | 92.09 |  |  |  |
| **Impurity F** | **Samples** | | **Added(ng)** | | **Found (ng)** | | **Recovery (%)** | | **Mean value (%)** | | **RSD (%)** |
|  | 80% level | | 1624.00 | | 1632.30 | | 100.51 | | 97.19 | | 5.83 |
|  |  |  | 1624.00 | | 1576.04 | | 97.05 | |  |  |  |
|  |  |  | 1624.00 | | 1731.18 | | 106.60 | |  |  |  |
|  |  |  | 1624.00 | | 1538.42 | | 94.73 | |  |  |  |
|  |  |  | 1624.00 | | 1519.10 | | 93.54 | |  |  |  |
|  |  |  | 1624.00 | | 1473.52 | | 90.73 | |  |  |  |
|  | 100% level | | 2030.00 | | 2102.77 | | 103.58 | | 100.94 | | 4.85 |
|  |  |  | 2030.00 | | 1939.73 | | 95.55 | |  |  |  |
|  |  |  | 2030.00 | | 2032.66 | | 100.13 | |  |  |  |
|  |  |  | 2030.00 | | 2191.61 | | 107.96 | |  |  |  |
|  |  |  | 2030.00 | | 1937.71 | | 95.45 | |  |  |  |
|  |  |  | 2030.00 | | 2089.45 | | 102.93 | |  |  |  |
|  | 120% level | | 2436.00 | | 2485.63 | | 102.04 | | 98.05 | | 3.35 |
|  |  |  | 2436.00 | | 2435.69 | | 99.99 | |  |  |  |
|  |  |  | 2436.00 | | 2373.12 | | 97.42 | |  |  |  |
|  |  |  | 2436.00 | | 2292.12 | | 94.09 | |  |  |  |
|  |  |  | 2436.00 | | 2300.42 | | 94.43 | |  |  |  |
|  |  |  | 2436.00 | | 2443.94 | | 100.33 | |  |  |  |
| **Impurity G** | | **Samples** | | **Added(ng)** | | **Found (ng)** | | **Recovery (%)** | **Mean value (%)** | **RSD (%)** | |
|  |  | 80% level | | 1619.20 | | 1511.66 | | 93.36 | 97.07 | 4.86 | |
|  |  |  |  | 1619.20 | | 1487.73 | | 91.88 |  |  |  |
|  |  |  |  | 1619.20 | | 1513.06 | | 93.45 |  |  |  |
|  |  |  |  | 1619.20 | | 1649.39 | | 101.86 |  |  |  |
|  |  |  |  | 1619.20 | | 1609.48 | | 99.40 |  |  |  |
|  |  |  |  | 1619.20 | | 1659.29 | | 102.48 |  |  |  |
|  |  | 100% level | | 2024.00 | | 1989.45 | | 98.29 | 95.03 | 4.62 | |
|  |  |  |  | 2024.00 | | 1922.95 | | 95.01 |  |  |  |
|  |  |  |  | 2024.00 | | 2006.33 | | 99.13 |  |  |  |
|  |  |  |  | 2024.00 | | 1824.15 | | 90.13 |  |  |  |
|  |  |  |  | 2024.00 | | 1991.28 | | 98.38 |  |  |  |
|  |  |  |  | 2024.00 | | 1805.70 | | 89.21 |  |  |  |
|  |  | 120% level | | 2428.80 | | 2502.49 | | 103.03 | 102.20 | 4.36 | |
|  |  |  |  | 2428.80 | | 2374.18 | | 97.75 |  |  |  |
|  |  |  |  | 2428.80 | | 2464.92 | | 101.49 |  |  |  |
|  |  |  |  | 2428.80 | | 2609.18 | | 107.43 |  |  |  |
|  |  |  |  | 2428.80 | | 2349.10 | | 96.72 |  |  |  |
|  |  |  |  | 2428.80 | | 2593.54 | | 106.78 |  |  |  |
| **Impurity H** | **Samples** | | **Added(ng)** | | **Found (ng)** | | **Recovery (%)** | | **Mean value (%)** | | **RSD (%)** |
|  | 80% level | | 1580.80 | | 1870.17 | | 109.66 | | 107.43 | | 1.96 |
|  |  |  | 1580.80 | | 1793.98 | | 103.72 | |  |  |  |
|  |  |  | 1580.80 | | 1810.32 | | 108.35 | |  |  |  |
|  |  |  | 1580.80 | | 1696.19 | | 108.69 | |  |  |  |
|  |  |  | 1580.80 | | 1763.27 | | 106.44 | |  |  |  |
|  |  |  | 1580.80 | | 1726.92 | | 107.72 | |  |  |  |
|  | 100% level | | 1976.00 | | 2179.05 | | 104.31 | | 103.63 | | 0.94 |
|  |  |  | 1976.00 | | 2057.42 | | 103.28 | |  |  |  |
|  |  |  | 1976.00 | | 2202.31 | | 104.28 | |  |  |  |
|  |  |  | 1976.00 | | 2145.48 | | 102.53 | |  |  |  |
|  |  |  | 1976.00 | | 2035.02 | | 102.56 | |  |  |  |
|  |  |  | 1976.00 | | 2159.65 | | 104.81 | |  |  |  |
|  | 120% level | | 2371.20 | | 2568.73 | | 104.37 | | 106.29 | | 1.78 |
|  |  |  | 2371.20 | | 2581.79 | | 105.17 | |  |  |  |
|  |  |  | 2371.20 | | 2633.10 | | 108.55 | |  |  |  |
|  |  |  | 2371.20 | | 2491.06 | | 104.78 | |  |  |  |
|  |  |  | 2371.20 | | 2417.36 | | 108.65 | |  |  |  |
|  |  |  | 2371.20 | | 2495.33 | | 106.22 | |  |  |  |

**8. Solution stability of impurities C ~ H at different time intervals**

| 0 h | 2 h |
| --- | --- |
| 4 h | 8 h |
| 12 h | 24 h |

| **Time（h）** | **0** | **2** | **4** | **8** | **12** | **24** | **Mean value** | **RSD（%）** |
| --- | --- | --- | --- | --- | --- | --- | --- | --- |
| Peak area of impurity C | 88 | 61 | 81 | 73 | 90 | 68 | 77 | 4.91 |
| Peak area of impurity D | 132585 | 126761 | 119025 | 121804 | 107668 | 114171 | 120336 | 7.37 |
| Peak area of impurity E | 6095 | 6060 | 6062 | 6170 | 6320 | 5990 | 6116 | 1.89 |
| Peak area of impurity F | 1822 | 1767 | 1725 | 1752 | 1667 | 1606 | 1723 | 4.47 |
| Peak area of impurity G | 39 | 34 | 36 | 33 | 33 | 34 | 35 | 6.67 |
| Peak area of impurity H | 1734 | 1727 | 1596 | 1792 | 1699 | 1786 | 1722 | 4.16 |

**9. Sensitivity of impurities C ~ H**

| Detection limit of impurity C    Detection limit of impurity D | Quantitation limit of impurity C    Quantitation limit of impurity D |
| --- | --- |
| Detection limit of impurity E | Quantitation limit of impurity E |
| Detection limit of impurity F | Quantitation limit of impurity F |
| Detection limit of impurity G | Quantitation limit of impurity G |
| Detection limit of impurity H | Quantitation limit of impurity H |
|  |  |

|  | **minimum detectable amount (pg)** | **detection limit (ppm)** | **minimum quantitative amount (pg)** | **quantitation limit (ppm)** |
| --- | --- | --- | --- | --- |
| Impurity C | 20 | 0.2 | 100 | 1.0 |
| Impurity D | 2.5 | 0.025 | 10 | 0.1 |
| Impurity E | 10 | 0.1 | 50 | 0.5 |
| Impurity F | 20 | 0.2 | 100 | 1.0 |
| Impurity G | 100 | 1.0 | 500 | 5.0 |
| Impurity H | 5 | 0.05 | 20 | 0.2 |

**10. MRM chromatograms of DMEBZ sample (Batch No: 20130323)**

**11. MRM chromatograms of DMEBZ sample (Batch No: 20130519)**

**12. MRM chromatograms of DMEBZ sample (Batch No: 20141001)**

**13. MRM chromatograms of DMEBZ sample (Batch No: 20180903)**

**14. MRM chromatograms of DMEBZ sample (Batch No: 20181001)**

**15. MRM chromatograms of DMEBZ sample (Batch No: 20181003)**
